# Association of the Innate Immunity and Inflammation Pathway with Advanced Prostate Cancer Risk

**DOI:** 10.1371/journal.pone.0051680

**Published:** 2012-12-14

**Authors:** Rémi Kazma, Joel A. Mefford, Iona Cheng, Sarah J. Plummer, Albert M. Levin, Benjamin A. Rybicki, Graham Casey, John S. Witte

**Affiliations:** 1 Department of Epidemiology and Biostatistics and Institute for Human Genetics, University of California San Francisco, San Francisco, California, United States of America; 2 Epidemiology Program, University of Hawai’i Cancer Center, University of Hawai’i, Honolulu, Hawai’i, United States of America; 3 Department of Preventive Medicine, Norris Comprehensive Cancer Center, Keck School of Medicine, University of Southern California, Los Angeles, California, United States of America; 4 Department of Biostatistics and Research Epidemiology, Henry Ford Health System, Detroit, Michigan, United States of America; National Cancer Institute, National Institutes of Health, United States of America

## Abstract

Prostate cancer is the most frequent and second most lethal cancer in men in the United States. Innate immunity and inflammation may increase the risk of prostate cancer. To determine the role of innate immunity and inflammation in advanced prostate cancer, we investigated the association of 320 single nucleotide polymorphisms, located in 46 genes involved in this pathway, with disease risk using 494 cases with advanced disease and 536 controls from Cleveland, Ohio. Taken together, the whole pathway was associated with advanced prostate cancer risk (P = 0.02). Two sub-pathways (intracellular antiviral molecules and extracellular pattern recognition) and four genes in these sub-pathways (*TLR1*, *TLR6*, *OAS1*, and *OAS2*) were nominally associated with advanced prostate cancer risk and harbor several SNPs nominally associated with advanced prostate cancer risk. Our results suggest that the innate immunity and inflammation pathway may play a modest role in the etiology of advanced prostate cancer through multiple small effects.

## Introduction

Prostate cancer is the most frequent and second most lethal cancer in men in the United States [1]. There is growing evidence that innate immunity and inflammation may play a role in prostate and other cancers [2], [3], [4]. Chronic inflammation could contribute to prostate cancer through several biological processes: the mutagenesis caused by oxidative stress; the remodeling of the extracellular matrix; the recruitment of immune cells, fibroblasts, and endothelial cells; or the induction of cytokines and growth factors contributing to a proliferative and angiogenic environment [2], [3], [5].

Compelling evidence supports a role for genes involved in the innate immunity and inflammation pathway in prostate cancer risk. Several genes harboring single nucleotide polymorphisms (SNPs) associated with prostate cancer risk have been identified, including: the pattern recognition receptors *MSR1*, *TLR1*, *TLR4*, *TLR5*, *TLR6*, and *TLR10* [6], [7], [8], [9], [10], [11], [12], [13], [14], [15], [16]; the antiviral gene *RNASEL* [9], [17], [18], [19], [20], [21]; the cytokines *MIC1*, *IL8*, *TNFα*, and *IL1RN* [13], [22], [23], [24], [25], [26]; and the pro-inflammatory gene *COX-2* [27], [28], [29], [30]. However, most of the previous studies have focused on individual SNPs or genes and very little is known about the impact of the overall innate immunity and inflammation pathway on developing more advanced prostate cancer.

Moreover, advanced prostate cancer cases have a higher public health burden than less advanced cases. Thus, identifying the components of the innate immunity and inflammatory process that increase the risk of advanced prostate cancer is of major importance.

To determine the role of innate immunity and inflammation in advanced prostate cancer, we investigated the association of 320 SNPs, located in 46 innate immunity and inflammation genes, with advanced prostate cancer risk. We undertook a comprehensive approach evaluating the association between disease risk and SNPs-sets pooled across the whole pathway, sub-pathways, and each gene, as well as individual SNPs.

## Materials and Methods

### Study Population

The case sample comprised 494 men with newly diagnosed, histologically confirmed prostate cancer, having either a Gleason score ≥7, a clinical stage ≥ T2c, or a serum Prostate Serum Antigen (PSA) at diagnosis >10 recruited from the major medical institutions in Cleveland, Ohio (Cleveland Clinic Foundation, University hospitals of Cleveland, and their affiliates) [31]. The control sample comprised 536 men frequency matched to cases by age (within 5 years), ethnicity, and medical institution, who underwent standard annual exams at the major medical institutions in Cleveland, and who did not have a previous history of non-skin cancer. The PSA was measured and found elevated in two controls. Further investigations lead us to reclassify them as advanced cases of prostate cancer, leaving us with a total of 494 advanced prostate cancer cases and 536 controls used here in our analyses. Approval for this study was obtained from the University Hospitals of Cleveland Institutional Review Board and the Cleveland Clinic Foundation Institutional Review Board, and written informed consent was obtained from all study participants. More details about this study population have been previously described [27], [29], [32], [33], [34], [35], [36].

### SNP Selection, Genotyping and Quality Control

We selected for study 46 candidate genes coding for proteins involved in innate immunity and inflammation, and further grouped these into 6 relevant biological sub-pathways using a previously proposed and published classification [37]. These sub-pathways were: 1) cytokine signaling (26 genes), 2) eicosanoid signaling (1 gene, i.e. *COX-2*), 3) extracellular pattern recognition (8 genes), 4) intracellular antiviral molecules (4 genes), 5) nuclear kappa-light chain-enhancer or activated B cell (NFKB) signaling (5 genes), and 6) selenoproteins (2 genes). The genes *SELS* and *SEP15* coding for selenoproteins were included because of their potential role in the control of the inflammatory response through regulation of cytokine production [38].

All SNPs located within and 2 kb upstream and 1 kb downstream of the sequence of the 46 candidate genes were identified through the International HapMap Project (www.hapmap.org) and the Genome Variation Server (SeattleSNPs) (http://gvs.gs.washington.edu/). Then, tagging SNPs were selected using the multimarker test criteria in the Tagger software program [39] to capture all common SNPs (minor allele frequency, MAF >0.05) with an r^2^≥0.8 across each candidate gene among European ancestry populations, forcing SNPs that are missense, non-synonymous and previously associated with prostate cancer to be included. Only one missense SNP was included for the genes *TLR3* and *IL6R*.

Moreover, 39 ancestry informative markers (AIMs) [40] were genotyped and principal component analysis was used to estimate genetic ancestry and account for population stratification [41]. The first principal component of this analysis distinguished African Americans from Caucasians and was used as an estimate of genetic ancestry.

Genotyping of the 330 SNPs was done on DNA extracted from blood samples using either the Illumina 500G BeadStation coupled with the GoldenGate assay, or the Applied Biosystems Taqman assay. Further quality control procedures were done separately for each of the two platforms and for each of the two ethnic groups (African-Americans and Caucasians). Ten SNPs that had a call rate <0.90, deviated from the expected Hardy-Weinberg proportions in both ethnic groups (P<0.01), or had a MAF below 0.01 in both ethnic groups were excluded. Individuals who had a call rate <0.90 were also excluded. After the quality control procedure, the data in the case-control sample used to test for association with risk of advanced prostate cancer included 320 tagging SNPs (Table S1) and 39 AIMs.

### Statistical Analysis

To analyze the whole set of 320 SNPs together, or sets of SNPs grouped by sub-pathways or genes, we used the SNP-set kernel-machine association test (SKAT v0.62) [42]. This method uses a logistic kernel-machine model, aggregating individual score test statistics of SNPs, and provides a global P-value for the set of variants tested that takes into account the joint effect of the SNPs in a given SNP-set and allows for incorporating the adjustment covariates: age, institution, and genetic ancestry. One advantage of SKAT over other pathway tests is that it adaptively finds the degrees of freedom of the test statistic in order to account for LD between genotyped SNPs. Assuming that each of the association coefficients for the *p* SNPs in a particular SNP-set (*β_Gp_*) independently follows an arbitrary distribution with mean 0 and variance *ψ*, testing the null hypothesis, *β_Gm_* = 0, is equivalent to testing *ψ* = 0 (*i.e.*, a variance-component test score done using the corresponding mixed model). For a case-control sample with *n* individuals sampled and *p* variants genotyped, **G** is the *n*×*p* matrix of genotypes, and **K** = **GG** ^T^ is an *n*×*n* linear kernel matrix, which defines the genetic similarity between all individuals for the *p* SNPs. The function that links each element of the matrix **K** to the genotypes **G** is the kernel function. To test for the association between the disease and the SNP-set, the variance-component score statistic Q follows a mixture of chi-square distributions.
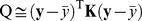
where, 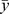 is the predicted mean of the vector of disease status values (**y**) under the null hypothesis, obtained by regressing **y** on the adjustment covariates only. For theses analyses, we used the linear kernel (equivalent to fitting the unconditional multivariate logistic regression) and the exact Davies method for computing p-values. Moreover, we tested for association of advanced prostate cancer risk with the 320 SNPs individually using unconditional multivariate logistic regression adjusting for age, institution, and genetic ancestry. Odds ratios (ORs), 95% confidence intervals (95% CI) and P-values were estimated using both co-dominant and log-additive models.

To adjust for genetic ancestry in all analyses, we included the first principal component of the principal component analysis of the 39 AIMs as covariate. Moreover, to identify SNPs with potential opposite effects in African Americans and Caucasians, we also stratified all analyses by reported ethnicity. Our strategy evaluated disease risk association at multiple levels of SNP groupings (whole set, sub-pathways, genes, and individual SNPs). To account for the multiple tests done while incorporating the correlation between SNPs and genotype coding, we used a permutation procedure to obtain the empirical distribution of statistical tests under the null hypothesis of no association with the set of SNPs or SNP. Then for each level of SNP groupings, we calculated a family-wise error rate by comparing the P-value of each test to the distribution of the minimum P-values obtained from 1000 permuted data sets. Reported P-values are two-sided and analyses were done using R v2.13.1 [43].

## Results

### Study Subject Characteristics

The case-control sample included 1,030 subjects whose average age at diagnosis or recruitment was 65.87 (SD: 8.46) years, and was comprised of 194 African Americans (18.8%) and 836 Caucasians (81.2%). Age and ethnicity were similarly distributed in advanced prostate cancer cases and controls (Table 1).

**Table 1:** Study characteristics of the advanced prostate cancer cases and controls.

	Cases		Controls		P-value of
	(*n* = 494)		(*n* = 536)		heterogeneity^a^
Age (year), mean (SD)	65.90	(8.34)	65.85	(8.54)	0.91
Ethnicity, *n* (%)					
African American	90	(18.2)	104	(19.4)	0.68
Caucasian	404	(81.8)	432	(80.6)	
Prostate cancer in first degree relative, *n* (%)^b^					
Negative	381	(77.3)	472	(88.9)	<2×10^−16^
Positive	112	(22.7)	59	(11.1)	
PSA at diagnosis (ng/mL), mean (SD)	14.38	(27.67)	1.74	(1.71)	<2×10^−16^
Categories of PSA at diagnosis, *n* (%)					
<4.0	25	(5.1)	–	–	
4.0–9.9	249	(50.4)	–	–	
10–19.9	152	(30.8)	–	–	
20–49.9	53	(10.7)	–	–	
>50	15	(3.0)	–	–	
Gleason score, *n* (%)					
≤6	74	(15.0)	–	–	
3+4	217	(43.9)	–	–	
4+3 or ≥8	203	(41.1)	–	–	
Clinical stage, *n* (%) b					
T1	306	(64.7)	–	–	
T2a-T2b	127	(26.8)	–	–	
T2c	15	(3.2)	–	–	
T3–T4	25	(5.3)	–	–	

a P-values obtained using either a Student t-test (quantitative coding) or a Chi-square test (qualitative coding).

b The sum of all categories does not add to the total due to missing data.

### Association with Advanced Prostate Cancer Risk

Taken together, the whole set of 320 SNPs in the innate immunity and inflammation pathway was significantly associated with advanced prostate cancer risk (P = 0.02). Of the 6 sub-pathways analyzed, the intracellular antiviral molecules and the extracellular pattern recognition sub-pathways were nominally associated with advanced prostate cancer risk (P = 0.02 for both) but not associated after correction for multiple testing (P = 0.12 and P = 0.11, respectively).

Interestingly, 4 genes in these 2 sub-pathways were also nominally associated with prostate cancer risk: *TLR1* and *TLR6* in the extracellular pattern recognition sub-pathway (P = 0.002 and P = 0.04, respectively), and *OAS1* and *OAS2* in the intracellular antiviral molecules sub-pathway (P = 0.015 and P = 0.019, respectively). In addition, *IFNGR1* in the cytokine signaling sub-pathway and *COX-2*, which is the sole member of the eicosanoid signaling sub-pathway represented in our data set, had nominal P-values of 0.006.and 0.044, respectively (Table 2). However, none of these associations are robust to correction for multiple testing (P = 0.10 for the association with *TLR1*).

**Table 2:** Association of the whole pathway, sub-pathways, and genes of innate immunity and inflammation with advanced prostate cancer risk.

SNP set	SNP count	P-value		
		Overall	African American	Caucasian
Inflammation and innate immunity	320	0.02	0.29	0.01
• Cytokine signaling (26 genes)	179	0.44	0.33	0.57
* IL10*	8	0.34	0.42	0.47
* IL12RB2*	11	0.75	0.89	0.61
* IL6R*	1	–a	–a	–a
* IL18R1*	16	0.11	0.09	0.31
* IL1B*	4	0.53	0.58	0.59
* IL1RN*	7	0.42	0.50	0.51
* IL12A*	4	0.12	0.66	0.13
* TGFBR2*	33	0.75	0.22	0.78
* IL2*	5	0.81	0.41	0.63
* IL8*	4	0.18	1	0.17
* IL12B*	6	0.45	0.59	0.46
* IL13*	4	0.84	0.11	0.95
* IL4*	4	0.41	0.23	0.60
* IL5*	1	–a	–a	–a
* IFNGR1*	5	0.006	0.16	0.009
* IL17*	8	0.41	0.56	0.21
* TNF/LTA*	11	0.72	0.44	0.92
* TGFBR1*	6	0.49	0.40	0.52
* IL18*	8	0.048	0.07	0.08
* IFNG*	6	0.19	0.20	0.40
* IL23A*	1	–a	–a	–a
* IL12RB1*	5	0.57	0.45	0.41
* MIC1*	6	0.94	0.10	0.51
* TGFB1*	4	0.22	0.08	0.68
* IFNGR2*	9	0.72	0.86	0.78
* MIF*	2	0.36	1	0.23
• Eicosanoid signaling (1 gene: *COX2*)	9	0.04	0.07	0.09
• Extracellular pattern recognition (8 genes)	56	0.02	0.12	0.01
* TLR5*	7	0.49	0.69	0.48
* TLR1*	7	0.002	0.09	0.004
* TLR10*	7	0.18	0.35	0.07
* TLR2*	8	0.63	0.28	0.37
* TLR3*	1	–a	–a	–a
* TLR6*	5	0.04	0.04	0.04
* MSR1*	16	0.37	0.09	0.36
* TLR4*	5	0.11	0.05	0.19
• Intracellular antiviral molecules (4 genes)	40	0.02	0.71	0.01
* RNASEL*	7	0.31	0.24	0.43
* EIF2AK2*	11	0.79	0.41	0.44
* OAS1*	5	0.015	0.92	0.01
* OAS2*	17	0.019	0.79	0.01
• NFKB^b^ signaling (5 genes)	27	0.32	0.04	0.48
* NFKB1*	10	0.70	0.49	0.58
* IKBKB*	7	0.18	0.46	0.13
* CHUK*	6	0.14	0.07	0.28
* RELA*	2	0.16	0.04	0.51
* NFKBIA*	2	0.67	0.24	0.72
• Selenoproteins (2 genes)	9	0.67	0.93	0.44
* SEP15*	5	0.37	0.74	0.21
* SELS*	4	0.95	0.86	0.94

a Genes with one SNP;

b NFKB: nuclear kappa-light chain-enhancer or activated B cell.

The results of the individual SNP analyses supported the findings obtained with the sub-pathway and gene sets. Indeed, most of the SNPs having a nominal association P-value below 0.01, belong to *TLR1*, *TLR6*, *OAS1*, *OAS2* or *COX-2* (Table 3). Moreover, many of the other SNPs in these genes have a p-value between 0.01 and 0.05 (Table S2). Interestingly, for all these SNPs, results indicate a protective effect of the minor allele with additive ORs between 0.73 and 0.77. But again, when correcting for multiple testing, these were no longer significant (P = 0.42 for the most significant association).

**Table 3:** Association of SNPs with advanced prostate cancer risk (P-value <0.01).

Gene (chromosome)	SNP			Overall		African Americans		Caucasians	
				OR (95% CI)	P-value	OR (95% CI)	P-value	OR (95% CI)	P-value
*TLR1* (4)	rs5743551		AA	1 (Ref)	–	1 (Ref)	–	1 (Ref)	–
			AG	0.75 (0.57, 0.99)	0.044	0.86 (0.11, 5.69)	0.876	0.73 (0.55, 0.97)	0.032
			GG	0.51 (0.34, 0.78)	**0.002**	0.52 (0.07, 3.35)	0.492	0.53 (0.32, 0.88)	0.014
			trend (G)	0.73 (0.60, 0.88)	**0.001**	0.64 (0.37, 1.1)	0.103	0.73 (0.59, 0.9)	**0.003**
*OAS2* (12)	rs1058480		CC	1 (Ref)	–	1 (Ref)	–	1 (Ref)	–
			CG	0.73 (0.56, 0.96)	0.026	1.44 (0.64, 3.29)	0.379	0.67 (0.5, 0.89)	**0.007**
			GG	0.54 (0.35, 0.82)	**0.005**	1.02 (0.12, 8.88)	0.988	0.5 (0.32, 0.78)	**0.002**
			trend (G)	0.73 (0.60, 0.89)	**0.001**	1.26 (0.65, 2.48)	0.495	0.7 (0.57, 0.85)	**3.8×10^−4^**
*OAS2* (12)	rs15895		GG	1 (Ref)	–	1 (Ref)	–	1 (Ref)	–
			GA	0.74 (0.57, 0.98)	0.034	1.44 (0.64, 3.29)	0.379	0.68 (0.51, 0.91)	**0.009**
			AA	0.54 (0.35, 0.82)	**0.005**	1.02 (0.12, 8.88)	0.988	0.5 (0.32, 0.78)	**0.002**
			trend (A)	0.74 (0.61, 0.89)	**0.002**	1.26 (0.65, 2.48)	0.495	0.7 (0.57, 0.85)	**4.6×10^−4^**
*TLR1* (4)	rs4833095		AA	1 (Ref)	–	1 (Ref)	–	1 (Ref)	–
			AG	0.76 (0.57, 1.00)	0.053	0.82 (0.1, 5.49)	0.840	0.75 (0.57, 1.01)	0.0545
			GG	0.53 (0.35, 0.81)	**0.003**	0.6 (0.07, 3.88)	0.586	0.5 (0.3, 0.83)	**0.008**
			trend (G)	0.74 (0.60, 0.90)	**0.002**	0.74 (0.42, 1.27)	0.274	0.73 (0.59, 0.9)	**0.003**
*TGFBR1* (9)	rs10512263		AA	1 (Ref)	–	1 (Ref)	–	1 (Ref)	–
			AG	2.05 (1.35, 3.16)	**0.001**	5.37 (1.28, 36.55)	0.0382	1.88 (1.21, 2.95)	0.006
			GG	0.92 (0.18, 4.20)	0.912	–	–	0.92 (0.18, 4.21)	0.914
			trend (G)	1.73 (1.19, 2.54)	**0.004**	5.37 (1.28, 36.55)	0.020	1.59 (1.08, 2.36)	0.019
*TLR6* (4)	rs5743795		GG	1 (Ref)	–	1 (Ref)	–	1 (Ref)	–
			GA	0.73 (0.55, 0.97)	0.027	1.3 (0.44, 3.89)	0.634	0.7 (0.52, 0.93)	0.016
			AA	0.53 (0.25, 1.05)	0.074	–	–	0.52 (0.25, 1.03)	0.068
			trend (A)	0.73 (0.58, 0.92)	**0.007**	1.3 (0.44, 3.89)	0.634	0.71 (0.56, 0.9)	**0.004**
*TLR6* (4)	rs5743794		GG	1 (Ref)	–	1 (Ref)	–	1 (Ref)	–
			GA	0.73 (0.55, 0.97)	0.029	1.3 (0.44, 3.91)	0.627	0.7 (0.52, 0.94)	0.017
			AA	0.53 (0.26, 1.05)	0.075	–	–	0.52 (0.25, 1.04)	0.070
			trend (A)	0.73 (0.58, 0.92)	**0.008**	1.3 (0.44, 3.91)	0.627	0.71 (0.56, 0.9)	**0.005**
*TLR1* (4)	rs5743618		GG	1 (Ref)	–	1 (Ref)	–	1 (Ref)	–
			GT	0.81 (0.61, 1.09)	0.15	–	–	0.79 (0.58, 1.06)	0.117
			TT	0.55 (0.37, 0.86)	**0.007**	–	–	0.55 (0.34, 0.89)	0.015
			trend (T)	0.76 (0.63, 0.93)	**0.008**	–	–	0.76 (0.61, 0.94)	**0.010**
*OAS2* (12)	rs1293767		GG			1 (Ref)	–	1 (Ref)	–
			GC	0.70 (0.54, 0.92)	0.012	1.22 (0.54, 2.74)	0.632	0.65 (0.49, 0.87)	**0.004**
			CC	0.67 (0.43, 1.05)	0.080	1.17 (0.05, 30.16)	0.914	0.64 (0.41, 1.01)	0.059
			trend (C)	0.77 (0.64, 0.94)	**0.010**	1.18 (0.58, 2.44)	0.643	0.75 (0.61, 0.92)	**0.005**
*COX-2* (1)	rs2745557		GG	1 (Ref)	–	1 (Ref)	–	1 (Ref)	–
			GA	0.68 (0.51, 0.90)	**0.007**	0.67 (0.33, 1.32)	0.249	0.67 (0.49, 0.91)	0.011
			AA	0.77 (0.36, 1.62)	0.496	–	–	0.93 (0.42, 2.04)	0.862
			trend (A)	0.74 (0.58, 0.94)	0.013	0.57 (0.3, 1.05)	0.074	0.77 (0.59, 0.99)	0.042
*OAS1* (12)	rs2285934		CC	1 (Ref)	–	1 (Ref)	–	1 (Ref)	–
			CA	0.64 (0.49, 0.85)	**0.002**	0.39 (0.17, 0.86)	0.021	0.7 (0.52, 0.95)	0.022
			AA	0.71 (0.48, 1.03)	0.074	0.83 (0.35, 1.91)	0.653	0.62 (0.39, 0.96)	0.033
			trend (A)	0.79 (0.66, 0.95)	0.014	0.97 (0.64, 1.47)	0.880	0.76 (0.62, 0.93)	**0.009**
*OAS2* (12)		rs13311	CC	1 (Ref)	–	1 (Ref)	–	1 (Ref)	–
			CA	1.03 (0.80, 1.33)	0.808	0.78 (0.44, 1.38)	0.403	1.11 (0.84, 1.47)	0.473
			AA	3.27 (1.55, 7.55)	**0.003**	–	–	3.42 (1.61, 7.93)	**0.002**
			trend (A)	1.21 (0.97, 1.51)	0.091	0.78 (0.44, 1.38)	0.402	1.32 (1.03, 1.68)	0.026
*TLR4* (9)		rs10759932	TT	1 (Ref)	–	1 (Ref)	–	1 (Ref)	–
			TC	0.79 (0.60, 1.05)	0.111	0.75 (0.41, 1.38)	0.360	0.81 (0.59, 1.11)	0.190
			CC	4.13 (1.63, 12.6)	**0.005**	5.67 (1.36, 38.68)	0.033	3.48 (1.05, 15.66)	0.061
			trend (C)	1.03 (0.81, 1.31)	0.827	1.23 (0.77, 1.99)	0.388	0.97 (0.73, 1.28)	0.822

Odds ratios (OR), 95% confidence intervals (95% CI) and P-values obtained using the unconditional multivariate logistic model adjusted on age, institution, and genetic ancestry (first Principal Component) for SNPs that had at least one of the three tests (heterozygous, rare homozygous or trend) with a P-value below 0.01.

Stratifying on ethnicity shows that most of the SNPs associated with the pathway, sub-pathways, and SNPs in the whole sample are also detected in Caucasians, which represents more than 80% of the sample, but not in African Americans (Tables 2, 3, and Table S2).

## Discussion

In this integrative analysis of the association of advanced prostate cancer risk with candidate genes involved in innate immunity and inflammation, we studied 320 SNPs and their joint effects across genes and sub-pathways. Taken as a whole, the overall innate immunity and inflammation pathway seems to be involved in advanced prostate cancer, but the individual elements of this association are not clear. Indeed, the whole set of 320 SNPs is significantly associated with advanced prostate cancer risk. However, none of the other evaluated associations with sub-pathways, genes, or individual SNPs were significant, when correcting for multiple testing by making permutation based estimates of the family-wise error rate.

Nonetheless, our results suggest that the extracellular pattern recognition, the intracellular antiviral molecules, and the eicosanoid signaling (ie, *COX-2*) could be components that play a potential role in advanced prostate cancer risk. Within those sub-pathways, 5 genes (*TLR1*, *TLR6*, *OAS1*, *OAS2*, and *COX-2*) were nominally associated with advanced prostate cancer risk. Moreover, these genes harbor several SNPs nominally associated with advanced prostate cancer risk.

*TLR1* and *TLR6* encode members of the toll-like receptor family. Their role is to recognize molecular patterns associated to infectious pathogens. Both are highly conserved from *Drosophilia* to humans and share structural and functional similarities. Moreover, TLR1 and TLR6 also share the ability to form a heterodimer with TLR2 to recognize peptidoglycan and lipoproteins on pathogens. TLR1 is specialized in the recognition of gram-positive bacteria. Several studies have reported prostate cancer associations with members of the toll-like receptor family [6], [12], [16]. In particular Sun et al. [12] observed multiple SNPs in strong linkage disequilibrium located on *TLR1*, *TLR6*, and *TLR10* associated with prostate cancer. In our dataset, we observed the same association with rs5743551on *TLR1* and rs5743795 on *TLR6*.

*OAS1* and *OAS2* encode for two enzymes of the 2–5A synthetase family, involved in the innate immune response to viral infections. These molecules are induced by interferons and activate RNase L (product of *RNASEL*) which degrades viral RNA and inhibits replication. Recently, Molinaro et al. [44] showed that RNA fractions of prostate cancer cell lines are able to bind and activate OAS molecules, whereas RNA fractions of normal prostate epithelial cells cannot. Also, viral infections, sexually transmitted diseases [45], [46], [47], [48], [49], [50], and infections with *Propionibacterium acnes*, a gram positive bacterium, [51], [52] have been suggested as triggers in prostate cancer. These infectious agents may be cleared after the acute infection. Nonetheless, these agents could possibly induce carcinogenesis through the activation of a chronic inflammatory response [53]. Only one study of the association between prostate cancer and *OAS1* was done on a smaller sample size and 3 SNPs different from our selection where an association with rs2660 was found [54].

*COX-2* encodes for the enzyme cyclooxygenase-2 (COX-2). COX-2 converts arachidonic acid to prostaglandin H2, which is a precursor for other tissue-specific inflammatory molecules (prostanoids). COX-2 was found to be overexpressed in prostate cancer tissue compared to the surrounding normal prostate tissue [55], [56], [57]. The association of genetic variants with prostate cancer risk has also been outlined in previous studies, including in the same dataset [27], [28], [29], [30], [58]. However, reports on the association between elevated expression of COX-2 in prostate cancer tissues and high Gleason score and recurrence of the disease have mixed results [59], [60], [61].

Our results are concordant with those reported by Zheng et al. [62] who studied 9,275 SNPs in 1,086 inflammation genes using 200 familial cases and 200 controls of Swedish origin. They observed a significant enrichment in the number of nominal associations observed, suggesting the role of multiple genes with modest effects. However, by using the SKAT, our study is the first analysis of SNP sets pooled across genes and sub-pathways within the innate immunity and inflammation pathway.

None of the SNPs or genes included in our study was reported in any of the genome-wide association studies of prostate cancer listed in the Catalog of Genome-Wide Association studies [63].

Nonetheless, our study has several limitations. First, the limited sample size, and thus limited power, could explain why the association with the whole set of genes is significant while none of the associations with the sub-pathways, genes, or SNPs are significant after correcting for multiple testing. With this sample, the minimum (or maximum for protective) odds ratio detectable with a power of 80% varies between 1.5 (or 0.67) and 2.19 (or 0.46) when the MAF varies between 0.5 and 0.05. Moreover, the limited sample size does not allow evaluating potential heterogeneous effects of variants by ethnicity or other covariates. Second, although a more stringent selection of cases would better describe the role of the innate immunity and inflammation pathway in advanced prostate cancer, it would decrease the sample size –and consequently the power– drastically. Third, our selection of SNPs cannot exclude the possibility for rare functional variants in these candidate genes to play a role in advanced prostate cancer risk. Third, although the SKAT method provides an ideal framework to test for association with sets of potentially correlated SNPs, it does not measure the increase in risk associated with variants in the set of SNPs.

In conclusion, this study furthers research into prostate cancer genetics by studying SNPs in a candidate pathway at multiple levels of information: whole pathway, sub-pathways, genes, and SNPs. Our results suggest that although it may not be central in the etiology of advanced prostate cancer, the innate immunity and inflammation pathway could play a role in prostate cancer through different genetic variants.

## Supporting Information

Table S1 Description of the 320 single nucleotide polymorphisms analyzed. A1: Minor (rarer) allele; A2: Other (frequent) allele; A1A1: Rarer homozygous genotype; A1A2: Heterozygous genotype; A2A2: Frequent homozygous genotype; MAF: Minor allele frequency; P_Hardy-Weinberg_: Hardy-Weinberg proportion adequacy test (chi-square test).
(XLSX)

Table S2 Association of all SNPs analyzed with advanced prostate cancer risk. The next 3 Excel sheets contain the results of the analyses for the whole sample (Overall) and stratified by ethnicities: African Americans and Caucasians. OR: Odds Ratio; 95% CI: 95% confidence interval; P-value: P-value of the Wald test of association of the heterozygote or rare homozygote genotypes compared to the common homozygote genotype or P-value of the allelic trend test.
(XLSX)

## References

[1] Jemal A, Siegel R, Xu J, Ward E (2010) Cancer statistics, 2010. CA Cancer J Clin 60: 277–300. 20610543 10.3322/caac.20073

[2] Coussens LM, Werb Z (2002) Inflammation and cancer. Nature 420: 860–867. 12490959 10.1038/nature01322 PMC2803035

[3] de Visser KE, Coussens LM (2005) The interplay between innate and adaptive immunity regulates cancer development. Cancer Immunol Immunother 54: 1143–1152. 15889249 10.1007/s00262-005-0702-5 PMC11032791

[4] de Visser KE, Eichten A, Coussens LM (2006) Paradoxical roles of the immune system during cancer development. Nat Rev Cancer 6: 24–37. 16397525 10.1038/nrc1782

[5] Hanahan D, Weinberg RA (2000) The hallmarks of cancer. Cell 100: 57–70. 10647931 10.1016/s0092-8674(00)81683-9

[6] Chen YC, Giovannucci E, Lazarus R, Kraft P, Ketkar S, et al (2005) Sequence variants of Toll-like receptor 4 and susceptibility to prostate cancer. Cancer Res 65: 11771–11778. 16357190 10.1158/0008-5472.CAN-05-2078

[7] Lindmark F, Jonsson BA, Bergh A, Stattin P, Zheng SL, et al (2004) Analysis of the macrophage scavenger receptor 1 gene in Swedish hereditary and sporadic prostate cancer. Prostate 59: 132–140. 15042613 10.1002/pros.10367

[8] Miller DC, Zheng SL, Dunn RL, Sarma AV, Montie JE, et al (2003) Germ-line mutations of the macrophage scavenger receptor 1 gene: association with prostate cancer risk in African-American men. Cancer Res 63: 3486–3489. 12839931

[9] Rennert H, Zeigler-Johnson CM, Addya K, Finley MJ, Walker AH, et al (2005) Association of susceptibility alleles in ELAC2/HPC2, RNASEL/HPC1, and MSR1 with prostate cancer severity in European American and African American men. Cancer Epidemiol Biomarkers Prev 14: 949–957. 15824169 10.1158/1055-9965.EPI-04-0637

[10] Seppala EH, Ikonen T, Autio V, Rokman A, Mononen N, et al (2003) Germ-line alterations in MSR1 gene and prostate cancer risk. Clin Cancer Res 9: 5252–5256. 14614006

[11] Sun J, Hsu FC, Turner AR, Zheng SL, Chang BL, et al (2006) Meta-analysis of association of rare mutations and common sequence variants in the MSR1 gene and prostate cancer risk. Prostate 66: 728–737. 16425212 10.1002/pros.20396

[12] Sun J, Wiklund F, Zheng SL, Chang B, Balter K, et al (2005) Sequence variants in Toll-like receptor gene cluster (TLR6-TLR1-TLR10) and prostate cancer risk. J Natl Cancer Inst 97: 525–532. 15812078 10.1093/jnci/dji070

[13] Xu J, Lowey J, Wiklund F, Sun J, Lindmark F, et al (2005) The interaction of four genes in the inflammation pathway significantly predicts prostate cancer risk. Cancer Epidemiol Biomarkers Prev 14: 2563–2568. 16284379 10.1158/1055-9965.EPI-05-0356

[14] Xu J, Zheng SL, Komiya A, Mychaleckyj JC, Isaacs SD, et al (2003) Common sequence variants of the macrophage scavenger receptor 1 gene are associated with prostate cancer risk. Am J Hum Genet 72: 208–212. 12471593 10.1086/345802 PMC378627

[15] Xu J, Zheng SL, Komiya A, Mychaleckyj JC, Isaacs SD, et al (2002) Germline mutations and sequence variants of the macrophage scavenger receptor 1 gene are associated with prostate cancer risk. Nat Genet 32: 321–325. 12244320 10.1038/ng994

[16] Zheng SL, Augustsson-Balter K, Chang B, Hedelin M, Li L, et al (2004) Sequence variants of toll-like receptor 4 are associated with prostate cancer risk: results from the CAncer Prostate in Sweden Study. Cancer Res 64: 2918–2922. 15087412 10.1158/0008-5472.can-03-3280

[17] Casey G, Neville PJ, Plummer SJ, Xiang Y, Krumroy LM, et al (2002) RNASEL Arg462Gln variant is implicated in up to 13% of prostate cancer cases. Nat Genet 32: 581–583. 12415269 10.1038/ng1021

[18] Nakazato H, Suzuki K, Matsui H, Ohtake N, Nakata S, et al (2003) Role of genetic polymorphisms of the RNASEL gene on familial prostate cancer risk in a Japanese population. Br J Cancer 89: 691–696. 12915880 10.1038/sj.bjc.6601075 PMC2376919

[19] Noonan-Wheeler FC, Wu W, Roehl KA, Klim A, Haugen J, et al (2006) Association of hereditary prostate cancer gene polymorphic variants with sporadic aggressive prostate carcinoma. Prostate 66: 49–56. 16114055 10.1002/pros.20320

[20] Rennert H, Bercovich D, Hubert A, Abeliovich D, Rozovsky U, et al (2002) A novel founder mutation in the RNASEL gene, 471delAAAG, is associated with prostate cancer in Ashkenazi Jews. Am J Hum Genet 71: 981–984. 12145743 10.1086/342775 PMC378554

[21] Wang L, McDonnell SK, Elkins DA, Slager SL, Christensen E, et al (2002) Analysis of the RNASEL gene in familial and sporadic prostate cancer. Am J Hum Genet 71: 116–123. 12022038 10.1086/341281 PMC384968

[22] Lindmark F, Zheng SL, Wiklund F, Balter KA, Sun J, et al (2005) Interleukin-1 receptor antagonist haplotype associated with prostate cancer risk. Br J Cancer 93: 493–497. 16106254 10.1038/sj.bjc.6602729 PMC2361575

[23] Lindmark F, Zheng SL, Wiklund F, Bensen J, Balter KA, et al (2004) H6D polymorphism in macrophage-inhibitory cytokine-1 gene associated with prostate cancer. J Natl Cancer Inst 96: 1248–1254. 15316060 10.1093/jnci/djh227

[24] McCarron SL, Edwards S, Evans PR, Gibbs R, Dearnaley DP, et al (2002) Influence of cytokine gene polymorphisms on the development of prostate cancer. Cancer Res 62: 3369–3372. 12067976

[25] Oh BR, Sasaki M, Perinchery G, Ryu SB, Park YI, et al (2000) Frequent genotype changes at -308, and 488 regions of the tumor necrosis factor-alpha (TNF-alpha) gene in patients with prostate cancer. J Urol 163: 1584–1587. 10751892

[26] Wu HC, Chang CH, Chen HY, Tsai FJ, Tsai JJ, et al (2004) p53 gene codon 72 polymorphism but not tumor necrosis factor-alpha gene is associated with prostate cancer. Urol Int 73: 41–46. 15263792 10.1159/000078803

[27] Cheng I, Liu X, Plummer SJ, Krumroy LM, Casey G, et al (2007) COX2 genetic variation, NSAIDs, and advanced prostate cancer risk. Br J Cancer 97: 557–561. 17609663 10.1038/sj.bjc.6603874 PMC2360347

[28] Panguluri RC, Long LO, Chen W, Wang S, Coulibaly A, et al (2004) COX-2 gene promoter haplotypes and prostate cancer risk. Carcinogenesis 25: 961–966. 14754878 10.1093/carcin/bgh100

[29] Reese AC, Fradet V, Witte JS (2009) Omega-3 fatty acids, genetic variants in COX-2 and prostate cancer. J Nutrigenet Nutrigenomics 2: 149–158. 19776642 10.1159/000235565 PMC2820568

[30] Shahedi K, Lindstrom S, Zheng SL, Wiklund F, Adolfsson J, et al (2006) Genetic variation in the COX-2 gene and the association with prostate cancer risk. Int J Cancer 119: 668–672. 16506214 10.1002/ijc.21864

[31] D’Amico AV, Whittington R, Malkowicz SB, Schultz D, Blank K, et al (1998) Biochemical outcome after radical prostatectomy, external beam radiation therapy, or interstitial radiation therapy for clinically localized prostate cancer. JAMA 280: 969–974. 9749478 10.1001/jama.280.11.969

[32] Cheng I, Plummer SJ, Neslund-Dudas C, Klein EA, Casey G, et al (2010) Prostate cancer susceptibility variants confer increased risk of disease progression. Cancer Epidemiol Biomarkers Prev 19: 2124–2132. 20651075 10.1158/1055-9965.EPI-10-0268 PMC2950095

[33] Hardin J, Cheng I, Witte JS (2011) Impact of consumption of vegetable, fruit, grain, and high glycemic index foods on aggressive prostate cancer risk. Nutr Cancer 63: 860–872. 21774611 10.1080/01635581.2011.582224 PMC3209415

[34] Liu X, Plummer SJ, Nock NL, Casey G, Witte JS (2006) Nonsteroidal antiinflammatory drugs and decreased risk of advanced prostate cancer: modification by lymphotoxin alpha. Am J Epidemiol 164: 984–989. 16931544 10.1093/aje/kwj294

[35] Ross PL, Cheng I, Liu X, Cicek MS, Carroll PR, et al (2009) Carboxypeptidase 4 gene variants and early-onset intermediate-to-high risk prostate cancer. BMC Cancer 9: 69. 19245716 10.1186/1471-2407-9-69 PMC2657151

[36] Rybicki BA, Neslund-Dudas C, Nock NL, Schultz LR, Eklund L, et al (2006) Prostate cancer risk from occupational exposure to polycyclic aromatic hydrocarbons interacting with the GSTP1 Ile105Val polymorphism. Cancer Detect Prev 30: 412–422. 17067754 10.1016/j.cdp.2006.09.004 PMC1769317

[37] Loza MJ, McCall CE, Li L, Isaacs WB, Xu J, et al (2007) Assembly of inflammation-related genes for pathway-focused genetic analysis. PLoS One 2: e1035. 17940599 10.1371/journal.pone.0001035 PMC2001184

[38] Kaushal N, Ghandi UH, Nelson SM, Narayan V, Prabhu KS (2012) Selenium and inflammation. In: Hartfield DL, Berry MJ, Gladyshev VN, editors. Selenium, its modelucal biology and role in human health. Third ed. New York, Dordrecht, Heidelberg, London: Springer. 443–456.

[39] de Bakker PI, Yelensky R, Pe’er I, Gabriel SB, Daly MJ, et al (2005) Efficiency and power in genetic association studies. Nat Genet 37: 1217–1223. 16244653 10.1038/ng1669

[40] Shriver MD, Parra EJ, Dios S, Bonilla C, Norton H, et al (2003) Skin pigmentation, biogeographical ancestry and admixture mapping. Hum Genet 112: 387–399. 12579416 10.1007/s00439-002-0896-y

[41] Price AL, Patterson NJ, Plenge RM, Weinblatt ME, Shadick NA, et al (2006) Principal components analysis corrects for stratification in genome-wide association studies. Nat Genet 38: 904–909. 16862161 10.1038/ng1847

[42] Wu MC, Kraft P, Epstein MP, Taylor DM, Chanock SJ, et al (2010) Powerful SNP-set analysis for case-control genome-wide association studies. Am J Hum Genet 86: 929–942. 20560208 10.1016/j.ajhg.2010.05.002 PMC3032061

[43] R Development Core Team (2011, Vienna, Austria) R: A Language and Environment for Statistical Computing. R Foundation for Statistical Computing.

[44] Molinaro RJ, Jha BK, Malathi K, Varambally S, Chinnaiyan AM, et al (2006) Selection and cloning of poly(rC)-binding protein 2 and Raf kinase inhibitor protein RNA activators of 2′,5′-oligoadenylate synthetase from prostate cancer cells. Nucleic Acids Res 34: 6684–6695. 17145707 10.1093/nar/gkl968 PMC1751551

[45] Baker LH, Mebust WK, Chin TD, Chapman AL, Hinthorn D, et al (1981) The relationship of herpesvirus to carcinoma of the prostate. J Urol 125: 370–374. 6259379 10.1016/s0022-5347(17)55039-9

[46] Grinstein S, Preciado MV, Gattuso P, Chabay PA, Warren WH, et al (2002) Demonstration of Epstein-Barr virus in carcinomas of various sites. Cancer Res 62: 4876–4878. 12208733

[47] Hayes RB, Pottern LM, Strickler H, Rabkin C, Pope V, et al (2000) Sexual behaviour, STDs and risks for prostate cancer. Br J Cancer 82: 718–725. 10682688 10.1054/bjoc.1999.0986 PMC2363322

[48] Hoffman LJ, Bunker CH, Pellett PE, Trump DL, Patrick AL, et al (2004) Elevated seroprevalence of human herpesvirus 8 among men with prostate cancer. J Infect Dis 189: 15–20. 14702148 10.1086/380568

[49] Samanta M, Harkins L, Klemm K, Britt WJ, Cobbs CS (2003) High prevalence of human cytomegalovirus in prostatic intraepithelial neoplasia and prostatic carcinoma. J Urol 170: 998–1002. 12913758 10.1097/01.ju.0000080263.46164.97

[50] Taylor ML, Mainous AG 3rd, Wells BJ (2005) Prostate cancer and sexually transmitted diseases: a meta-analysis. Fam Med 37: 506–512. 15988645

[51] Cohen RJ, Shannon BA, McNeal JE, Shannon T, Garrett KL (2005) Propionibacterium acnes associated with inflammation in radical prostatectomy specimens: a possible link to cancer evolution? J Urol 173: 1969–1974. 15879794 10.1097/01.ju.0000158161.15277.78

[52] Lee JC, Muller CH, Rothman I, Agnew KJ, Eschenbach D, et al.. (2003) Prostate biopsy culture findings of men with chronic pelvic pain syndrome do not differ from those of healthy controls. J Urol 169: 584–587; discussion 587–588. 10.1097/01.ju.0000045673.02542.7a 12544312

[53] Platz EA, De Marzo AM (2004) Epidemiology of inflammation and prostate cancer. J Urol 171: S36–40. 14713751 10.1097/01.ju.0000108131.43160.77

[54] Mandal S, Abebe F, Chaudhary J (2011) 2′-5′ oligoadenylate synthetase 1 polymorphism is associated with prostate cancer. Cancer 117: 5509–5518. 21638280 10.1002/cncr.26219 PMC3167978

[55] Gupta S, Srivastava M, Ahmad N, Bostwick DG, Mukhtar H (2000) Over-expression of cyclooxygenase-2 in human prostate adenocarcinoma. Prostate 42: 73–78. 10579801 10.1002/(sici)1097-0045(20000101)42:1<73::aid-pros9>3.0.co;2-g

[56] Kirschenbaum A, Klausner AP, Lee R, Unger P, Yao S, et al (2000) Expression of cyclooxygenase-1 and cyclooxygenase-2 in the human prostate. Urology 56: 671–676. 11018637 10.1016/s0090-4295(00)00674-9

[57] Lee LM, Pan CC, Cheng CJ, Chi CW, Liu TY (2001) Expression of cyclooxygenase-2 in prostate adenocarcinoma and benign prostatic hyperplasia. Anticancer Res 21: 1291–1294. 11396201

[58] Hedelin M, Chang ET, Wiklund F, Bellocco R, Klint A, et al (2007) Association of frequent consumption of fatty fish with prostate cancer risk is modified by COX-2 polymorphism. Int J Cancer 120: 398–405. 17066444 10.1002/ijc.22319

[59] Cohen BL, Gomez P, Omori Y, Duncan RC, Civantos F, et al (2006) Cyclooxygenase-2 (COX-2) expression is an independent predictor of prostate cancer recurrence. Int J Cancer 119: 1082–1087. 16557596 10.1002/ijc.21749

[60] Wang W, Bergh A, Damber JE (2005) Cyclooxygenase-2 expression correlates with local chronic inflammation and tumor neovascularization in human prostate cancer. Clin Cancer Res 11: 3250–3256. 15867220 10.1158/1078-0432.CCR-04-2405

[61] Zha S, Gage WR, Sauvageot J, Saria EA, Putzi MJ, et al (2001) Cyclooxygenase-2 is up-regulated in proliferative inflammatory atrophy of the prostate, but not in prostate carcinoma. Cancer Res 61: 8617–8623. 11751373

[62] Zheng SL, Liu W, Wiklund F, Dimitrov L, Balter K, et al (2006) A comprehensive association study for genes in inflammation pathway provides support for their roles in prostate cancer risk in the CAPS study. Prostate 66: 1556–1564. 16921508 10.1002/pros.20496

[63] Hindorff LA, Sethupathy P, Junkins HA, Ramos EM, Mehta JP, et al (2009) Potential etiologic and functional implications of genome-wide association loci for human diseases and traits. Proc Natl Acad Sci U S A 106: 9362–9367. 19474294 10.1073/pnas.0903103106 PMC2687147

